# Office blood pressure versus ambulatory blood pressure measurement in childhood obesity

**DOI:** 10.1186/s12887-023-04010-4

**Published:** 2023-04-29

**Authors:** Laila B van der Heijden, Jaap W. Groothoff, Edith JM Feskens, Arieke J Janse

**Affiliations:** 1 Department of Pediatrics, Hospital Gelderse Vallei, P.O. Box 9025, Ede, 6710 HN The Netherlands; 2 Department of Pediatric Nephrology, Emma Children’s Hospital Amsterdam University Medical Center, University of Amsterdam, Meibergdreef 9, Amsterdam, 1105 AZ The Netherlands; 3 Division of Human Nutrition and Health, Wageningen University, P.O. Box 17, Wageningen, 6700 AA The Netherlands

**Keywords:** Pediatric obesity, Hypertension, Adolescent, Child, Masked hypertension, White coat hypertension

## Abstract

**Background:**

The prevalence of obesity-related co-morbidities is rising parallel to the childhood obesity epidemic. High blood pressure (BP), as one of these co-morbidities, is detected nowadays at increasingly younger ages. The diagnosis of elevated BP and hypertension, especially in the childhood population, presents a challenge to clinicians. The added value of ambulatory blood pressure measurement (ABPM) in relation to office blood pressure (OBP) measurements in obese children is unclear. Furthermore, it is unknown how many overweight and obese children have an abnormal ABPM pattern. In this study we evaluated ABPM patterns in a population of overweight and obese children and adolescents, and compared these patterns with regular OBP measurements.

**Methods:**

In this cross-sectional study in overweight or obese children and adolescents aged 4–17 years who were referred to secondary pediatric obesity care in a large general hospital in The Netherlands, OBP was measured during a regular outpatient clinic visit. Additionally, all participants underwent a 24-hour ABPM on a regular week-day. Outcome measures were OBP, mean ambulatory SBP and DBP, BP load (percentage of readings above the ambulatory 95th blood pressure percentiles), ambulatory BP pattern (normal BP, white-coat hypertension, elevated BP, masked hypertension, ambulatory hypertension), and BP dipping.

**Results:**

We included 82 children aged 4–17 years. They had a mean BMI Z-score of 3.3 (standard deviation 0.6). Using ABPM, 54.9% of the children were normotensive (95% confidence interval 44.1–65.2), 26.8% had elevated BP, 9.8% ambulatory hypertension, 3.7% masked hypertension, and 4.9% white-coat hypertension. An isolated night-time BP load > 25% was detected in almost a quarter of the children. 40% of the participants lacked physiologic nocturnal systolic BP dipping. In the group of children with normal OBP, 22.2% turned out to have either elevated BP or masked hypertension on ABPM.

**Conclusions:**

In this study a high prevalence of abnormal ABPM patterns in overweight or obese children and adolescents was detected. Additionally, OBP poorly correlated with the child’s actual ABPM pattern. Herewith, we emphasized the usefulness of ABPM as an important diagnostic tool in this population.

## Background

As a consequence of the global obesity epidemic, prevalence rates of obesity-related co-morbidities such as elevated blood pressure (BP) in children are also increasing [1]. Hypertension prevalences up to almost 25% are found in overweight children and adolescents [2–4]. Although the cardiovascular sequelae of hypertension are clinically obvious in adulthood, the consequences of high BP in children and adolescents are usually less clear on first sight. Hypertension in children and adolescents is associated with the development of early, often subclinical, hypertensive target-organ damage (TOD) including increased carotid intima-media thickness, left ventricular hypertrophy, insulin resistance, and renal damage [5–8]. In addition, numerous studies have shown that high BP in childhood increases the risk for adult hypertension and metabolic syndrome [9–12].

The diagnosis of elevated BP and hypertension depends on an accurate BP measurement, which can present a challenge to the clinician. Ambulatory 24-hour blood pressure measurement (ABPM) is strongly recommended for the diagnosis and management of hypertension [13–16]. ABPM allows a more representative observation of BP thoughout day and night compared to office blood pressure (OBP) measurements as well as assessment of the circadian and even ultradian BP variability. ABPM is useful to detect white-coat hypertension, masked hypertension, and nocturnal hypertension [17–19]. White-coat hypertension and masked hypertension are known to be more prevalent in obese compared to lean pediatric populations [3]. Furthermore, ABPM has been shown in children to be more predictive of end-organ damage [20]. However, healthcare workers may hesitate to perform ABPM, for example because they do not want to burden the patient when they think it is not necessary, especially in the childhood clinic. Furthermore, an adequate APBM could be difficult to obtain.

This study was conducted to evaluate ABPM patterns in a population of overweight and obese children and adolescents referred to our pediatric outpatient clinic, and to compare ABPM patterns with regular OBP measurements, with the aim to show the additional value of ABPM in this population. Our hypothesis is that the prevalence of abnormal ABPM patterns, including white-coat hypertension and masked hypertension, is substantial in childhood obesity. Furthermore, we expect a high prevalence of abnormal circadian variability in this population.

## Methods

### Study design and participants

A retrospective chart cross-sectional study was performed using data of overweight or obese children and adolescents aged 4–17 years who were referred to the outpatient clinic of Hospital Gelderse Vallei Ede between April 2015 and July 2017. Inclusion criteria were (1) overweight or obesity determined according to the internationally used BMI cut-off points as proposed by Cole et al [21]. with no syndromal or endocrine underlying problem, (2) a height of ≥ 120 cm [20], and (3) no (past) treatment with medications influencing cardiovascular function, body composition, lipid, or glucose metabolism.

This study was labeled as a non WMO (Wet medisch wetenschappelijk onderzoek met mensen, the Medical- Research Involving Human Subjects Act) study, which is an observational study in which no action or behavior is imposed to the participants. The studywas approved by the institutional review board of Hospital Gelderse Vallei Ede.

### Anthropometric measurements

Trained staff measured children’s weight in underwear using an electronic calibrated scale (Seca 761), and height without shoes using a stadiometer (Holtain Ltd., UK). Age and sex-specific BMI Z-scores were calculated using Dutch growth curves of 2010 based on the LMS analysis method [22]. In the remaining sections of this paper, the term overweight will be used to indicate both overweight and obesity.

Waist circumference was used as a marker of central adiposity and measured with a flexible tape to the nearest 0.1 cm at umbilicus height.

### BP measurements

#### Office blood pressure

Right arm OBP (during outpatient clinic visit) was measured in a supine position with an automated BP monitor (Welch Allyn VSM 300, USA) after 5 min of rest, using an appropriate cuff size. A minimum of two OBP measurements was taken to obtain two values not differing > 5 mmHg. The mean of these two measurements was used for data analysis. Reference values according to the recently updated Clinical Practice Guideline on BP in children were used [23].

#### Ambulatory blood pressure measurement (ABPM)

All participants underwent a 24-hour ABPM on a regular week-day using a SpaceLabs Ultralite 90217-1Q monitor, using an appropriate cuff size around the participant’s non-dominant hand. Participants were instructed to record activity, sleep, and wake times in a diary, and to continue their normal activities but refrain from contact sports and vigorous exercise. Readings were automatically taken every 15 min (waking hours) and every 60 min (night-time). Measurements were repeated twice at 2-minute interval if systolic or diastolic BP was > 95th percentile of reference population.

ABPM data were downloaded using the manufacturer’s software Spacelabs Medical ABP Report Management System version 2.00.09, firmware version 03.02.15 and Sentinel Cardiology Information Management System. Only ABPM profiles with at least 10 valid recordings during daytime and five during night-time were accepted for analysis. Values that fall outside of the following range were discarded: systolic BP 60–220 mmHg, diastolic BP 35–120 mmHg, heart rate 40–180 bpm, pulse pressure 40–120 mmHg [20].

Combining systolic and diastolic BP readings with the corresponding time of measurements, the variables were calculated as presented in Table 1. A combination of OBP [23], mean ambulatory BP, and BP load was used to categorize ABPM results as normal or abnormal, using the suggested scheme for staging of ambulatory BP levels in children as presented by Flynn et al. (Table 1) [20].

**Table 1:** Overview of the ABPM variables calculated in this study, and the ABPM pattern definitions used

		**Background information**	**Definitions**	**Used cut-off values**
**OBP**				1
**ABPM variables**				
	Mean 24-hour, daytime and night-time ambulatory SBP and DBP			95th percentile mean ambulatory SBP and DBP cut-offs as specified in 2
	Indexation of BP	To control for differences in age and body size seen across a typical pediatric cohort.	Indexed blood pressure = average measured ambulatory blood pressure value / 95th percentile ambulatory blood pressure; Sorof et al., 2001	
	BP load during the 24-hour period, daytime and night-time period (both SBP and DBP)	The amount of time that a subject’s SBP or DBP exceeds normal values. Mathematically it depends on both average BP levels and distribution of BP readings.(Wühl et al., 2002; Soergel et al., 1997)	Percentage of readings above the ambulatory 95th blood pressure percentiles as specified in 2.	Normal BP load: <25%.(Sorof et al., 2002; White et al., 1989)
	BP dipping		Percent decline in SBP and DBP during sleep ([mean daytime BP-mean night-time BP] / mean daytime BP x 100).	Normal dipping: ≥10% decline in mean systolic and diastolic ambulatory blood pressure levels from day to night.(Wilson et al., 1999)
**ABPM patterns** (according to the suggested scheme for staging of ambulatory blood pressure levels in children as presented in 2)*				
		**OBP**	**Mean ambulatory SBP or DBP**	**SBP or DBP load**
	**Normal blood pressure**	< 90th percentile	< 95th percentile	< 25%
	**White-coat hypertension**	≥ 95th percentile	< 95th percentile	< 25%
	**Elevated blood pressure**	≥ 90th percentile or > 120/80 mm Hg	< 95th percentile	≥ 25%
	**Masked hypertension**	< 95th percentile	> 95th percentile	≥ 25%
	**Ambulatory hypertension**	> 95th percentile	> 95th percentile	25–50%, > 50% severe ambulatory hypertension

For some participants (n = 18) no specific ambulatory BP classification could be assigned based on the suggested scheme, i.e. (1) participants with normal OBP, normal mean ambulatory BP, but elevated load (n = 9), or (2) participants with OBP 90-95 h percentile, normal mean ambulatory BP, and normal load (n = 6). These subjects were evaluated on a case-by-case basis, as suggested by Flynn et al., taking into account the increased risk of cardiovascular disease (CVD) in subjects with overweight and the clinical relevance of elevated BP load. Those with unclassified AHA BP parameters in the first group were classified as ‘elevated BP’, subjects in the second group as ‘normal BP’. Next, one participant with normal OBP, normal mean ambulatory BP, and elevated night-time BP load (> 50%) with low systolic and diastolic BP dipping (respectively 4.9 and 0.4%) was classified as masked hypertension. Two participants with OBP in the ‘elevated BP’ range, normal mean ambulatory BP and elevated night-time BP load were classified as ‘elevated BP’.

1 Clinical Practice Guideline for Screening and Management of High Blood Pressure in Children and Adolescents; Flynn et al., 2017

2 Update: Ambulatory Blood Pressure Monitoring in Children and Adolescents, a Scientific Statement from the American Heart Association (AHA); Flynn et al., 2014

ABPM, ambulatory blood pressure measurement; BP, blood pressure; DBP, diastolic blood pressure; SBP, systolic blood pressure

* In line with the Clinical Practice Guideline the term ‘prehypertension’ was replaced with ‘elevated BP’ in subjects with prehypertension according to this classification scheme.

### Laboratory measurements

After an overnight fast, serum glucose, glycated haemoglobin (HbA1c), insulin levels, and lipid profiles were determined. All participants underwent a 2-h oral glucose tolerance test with a 1.75 gram glucose dose per kilogram bodyweight (maximum of 75 gram). Glucose tolerance status was determined according to the American Diabetes Association 2018 criteria. Insulin resistance was analysed using the formula [fasting insulin (mIU/L) x fasting glucose (mmol/L)]/22.5. Homeostasis model assessment of insulin resistance (HOMA-IR) cut-off values were used as proposed by Kurtoglu et al [24]. Dyslipidemia was defined as elevated total cholesterol and/or elevated LDL cholesterol and/or HDL cholesterol below cut-off and/or elevated triglycerides, using the age-specific reference values obtained from the Dutch guideline for childhood obesity and cardiovascular risk management [25]. For the diagnosis of metabolic syndrome an adjusted definition was used; the presence of central obesity (waist circumference ≥ 90th percentile) plus any of the other four components of metabolic syndrome: elevated triglycerides, HDL cholesterol below cut-off, disordered glucose metabolism (prediabetes or diabetes)[26], or abnormal OBP [23].

### Statistical analysis

SPSS 19.0 (IBM SPSS Statistics Inc., Chicago, IL) statistical package was used to analyze the data. Normalcy of the data was determined with Skewness and Kurtosis tests. Mann-Whitney U tests (for continuous variables) and Pearson Chi-Square tests (for categorical variables) were used to compare between dippers and non-dippers and between the different ABPM patterns with normal BP as the reference category. P < 0.05 was considered statistically significant.

### Patient and public involvement

Patients and the public were not involved in the design, conduct, reporting or dissemination plans of this research.

## Results

Our 82 participants were aged 4–17 years, and 39% of them were boys (Table 2). Ten participants were classified as overweight, the remaining 72 (88%) were obese. 60% of the participants presented with at least one obesity-related comorbidity. No participant was treated with antihypertensive medication at the time of the ABPM. The average amount of 24-hour ABPM readings per patient was 39 (standard deviation 6).

**Table 2:** Participant characteristics of the total study population, and for the ABPM categories separately

				ABPM				
		Total group (n = 82)		Normal BP (n = 45)	WCH (n = 4)	Elevated BP (n = 22)	MH (n = 3)	Ambulatory HTN (n = 8)
Male		32 (39.0)		20 (44.4)	3 (75.0)	6 (27.3)	1 (33.3)	2 (25.0)
Age		11.8 (8.8, 14.6)		11.5 (8.7, 14.6)	8.4 (5.9, 11.5)	12.9 (9.0, 14.9)	10.8 (6.8, NA)	9.9 (9.1, 16.3)
Ethnicity^‡^								
	Dutch / Western immigrant	66 (81.5)		37 (82.2)	3 (75.0)	17 (77.2)	3 (100.0)	6 (75.0)
	Non-western immigrant	16 (19.5)		8 (17.8)	1 (25.0)	5 (22.7)	0 (0.0)	2 (25.0)
BMI Z-score		3.3 (2.8, 3.6)		3.1 (2.8, 3.5)	3.1 (2.8, 3.6)	3.5 (2.9, 3.8)	2.7 (2.4, NA)	3.8 (3.1, 4.3)^b^
Glucose metabolism								
	Prediabetes*	8 (10.4)		4 (9.5)	0 (0.0)	3 (14.3)	0 (0.0)	1 (12.5)
	Elevated HOMA-IR^†^	33 (44.0)		16 (39.0)	0 (0.0)	9 (45.0)	0 (0.0)	8 (100.0)^a^
Lipid profile								
	Elevated total cholesterol*	3 (3.9)		2 (4.8)	0 (0.0)	1 (4.8)	0 (0.0)	0 (0.0)
	Elevated LDL-C*	1 (1.2)		1 (2.4)	0 (0.0)	0 (0.0)	0 (0.0)	0 (0.0)
	HDL-C below cut-off*	10 (13.0)		4 (9.5)	1 (25.0)	3 (14.3)	0 (0.0)	2 (25.0)
	Elevated triglycerides*	8 (10.4)		3 (7.1)	0 (0.0)	3 (14.3)	0 (0.0)	2 (25.0)
MetS**		11 (13.4)		2 (5.1)	1 (25.0)	6 (30.0)^a^	0 (0.0)	2 (28.6)^b^

Number (%), except for age and BMI Z-score (median, interquartile range)

* Total group: n = 77; normal BP: n = 42; elevated BP: n = 21; MH: n = 2

** Total group: n = 79; normal BP: n = 39; elevated BP: n = 20; MH: n = 2; (severe) ambulatory HTN: n = 7.

† Total group: n = 75; normal BP: n = 41; elevated BP: n = 20; MH: n = 2.

‡ Children whose parents were born outside the Netherlands were identified as immigrants (even if the child was of Dutch nationality). If both parents were born in the Netherlands, the child was classified as native Dutch. Western immigrants originated from Europe (excluding Turkey), North America, Oceania, Indonesia or Japan. Non-western immigrants originated from Africa, South America, Asia (excluding Indonesia and Japan) or Turkey.

a p < 0.01 compared to normal BP, using Mann-Whitney U test for continuous variables and Pearson Chi-Square for categorical variables

b p < 0.05 compared to normal BP, using Mann-Whitney U test for continuous variables and Pearson Chi-Square for categorical variables

ABPM, ambulatory blood pressure measurement; BMI, body mass index; BP, blood pressure; HDL-C, high-density lipoprotein cholesterol; HTN, hypertension; HOMA-IR, homeostasis model assessment of insulin resistance; LDL-C, low-density lipoprotein cholesterol; MetS, metabolic syndrome; MH, masked hypertension; WCH, white-coat hypertension

Based on OBP measurements 54.9% (95% confidence interval [CI] 44.1–65.2; n = 45) of the participants were normotensive, 19.5% had elevated BP, 19.5% classified as stage 1 hypertension, and 6.1% were classified as stage 2 hypertension (Table 3). 22% of the participants had an office systolic BP index ≥ 1.0, 9% an office diastolic BP index ≥ 1.0.

**Table 3:** Summary of blood pressure characteristics according to ABPM classification

		**ABPM**				
	Total group (n = 82)	Normal BP (n = 45)	**WCH (n = 4)**	**Elevated BP (n = 22)**	**MH (n = 3)**	**Ambulatory HTN (n = 8)**
OBP						
Normal BP	45 (54.9)	35 (77.8)	0 (0.0)	9 (40.9)	1 (33.3)	0 (0.0)
Elevated BP	16 (19.5)	9 (20.0)	0 (0.0)	5 (22.7)	2 (66.7)	0 (0.0)
Stage 1 HTN	16 (19.5)	1 (2.2)	3 (75.0)	7 (31.8)	0 (0.0)	5 (62.5)
Stage 2 HTN	5 (6.1)	0 (0.0)	1 (25.0)	1 (4.5)	0 (0.0)	3 (37.5)
24-hour SBP						
Median (IQR)	109 (104, 116)	106 (101, 112)	106 (97, 113)	111 (107, 119)	123 (110, NA)	124 (114, 130)
BP index, median (IQR)	0.88 (0.84, 0.93)	0.86 (0.82, 0.88)	0.87 (0.83, 0.92)	0.92 (0.87, 0.95)	0.96 (0.94, NA)	1.02 (0.98, 1.05)
BP index ≥ 1.0, n (%)	7 (8.5)	0 (0.0)	0 (0.0)	0 (0.0)	1 (33.3)	6 (75.0)
BP load (%), median (IQR)	8.8 (3.6, 25.4)	6.1 (0.0, 14.2)	4.2 (1.0, 13.0)	16.2 (6.7, 27.9)	28.6 (17.2, NA)	58.1 (44.4, 72.9)
BP load > 25%, n (%)	20 (24.4)	3 (6.7)	0 (0.0)	8 (36.4)	2 (66.7)	7 (87.5)
Dipping, median (IQR)	11 (7, 15)	12.8 (8.5, 17.1)	9.8 (7.5, 11.1)	10.4 (4.7, 13.4)	5.2 (4.9, NA)	6.8 (0.8, 12.1)
Dipping < 10%, n (%)	33 (40.2)	14 (31.1)	2 (50.0)	11 (50.0)	2 (66.7)	4 (50.0)
24-hour DBP						
Median (IQR)	65 (61, 69)	63 (60, 66)	62 (56, 66)	67 (64, 71)	73 (64, NA)	76 (69, 81)
BP index, median (IQR)	0.86 (0.81, 0.91)	0.82 (0.79, 0.87)	0.83 (0.74, 0.85)	0.89 (0.84, 0.93)	0.96 (0.88, NA)	0.99 (0.91, 1.07)
BP index ≥ 1.0, n (%)	4 (4.9)	0 (0.0)	0 (0.0)	0 (0.0)	1 (33.3)	3 (37.5)
BP load (%), median (IQR)	15.5 (6.3, 25.7)	11.9 (4.1, 20.5)	8.8 (3.8, 21.3)	17.7 (13.1, 30.6)	37.9 (16.7, NA)	53.6 (22.3, 73.6)
BP load > 25%, n (%)	22 (26.8)	6 (13.3)	0 (0.0)	9 (40.9)	2 (66.7)	5 (62.5)
Dipping, median (IQR)	19 (14, 22)	19.4 (14.3, 24.5)	16.2 (12.3, 23.0)	17.9 (14.7, 20.7)	19.4 (0.4, NA)	14.6 (7.0, 24.0)
Dipping < 10%, n (%)	9 (11.0)	3 (6.7)	0 (0.0)	3 (13.6)	1 (33.3)	2 (25.0)
Non-dipper, n (%)	34 (41.5)	14 (31.1)	2 (50.0)	12 (54.5)	2 (66.7)	4 (50.0)

Number (%), except for age, BMI, BMI Z-score, WC, WC Z-score (median, IQR).

BP, blood pressure; HTN, hypertension; IQR, interquartile range; MH, masked hypertension; WCH, white-coat hypertension

The ABPM patterns also showed that 54.9% (95% CI 44.1–65.2) of participants had normal BP. However, this group differed from the OBP normotensive group (Table 3). Ten of the 45 participants (22.2%) with normal OBP turned out to have either elevated BP (n = 9) or masked hypertension (n = 1) on ABPM (Table 3). Of the 37 participants with abnormal OBP, ten had normal BP on APBM (27.0%). Eight of the 21 participants (38%) with OBP hypertension were confirmed to have ambulatory hypertension. The others were diagnosed with elevated BP (n = 8) or white-coat hypertension (n = 4). Of the 16 participants with elevated BP in the outpatient clinic, 56% had normal ABPM results, 13% had masked hypertension, and in 31% of the participants elevated BP was confirmed with ABPM. The correlation coefficient for the relationship between indexed office systolic BP (as a proxy of hypertensive status based on OBP) and indexed mean daytime systolic BP (hypertensive status based on ABPM) was 0.39.

Using ABPM, a BP load > 25% was found in 24.4–26.8% of the participants during the whole 24-hour period. During night-time more often a BP load > 25% was detected than during daytime. 24% of all cases (n = 20) showed an isolated night-time BP load > 25% with normal daytime ABPM. Up to 40% of the participants lacked physiologic nocturnal systolic BP dipping.

No significant differences in terms of age and gender were observed between the different ABPM categories (Table 2). Participants with ambulatory hypertension had a significantly higher BMI Z-score (3.8, interquartile range [IQR] 3.1–4.3) compared to the normal BP group (BMI Z-score 3.1, IQR 2.8–3.5). Prediabetes was detected in 8 participants (11.1%); 50% of them had elevated BP or ambulatory hypertension.

The prevalence of metabolic syndrome was significantly higher in participants with elevated BP and ambulatory hypertension than in participants with normal ABPM. Of note, metabolic syndrome was significantly more prevalent in non-dippers when compared to dippers (29.6% versus 6.7%, respectively, p = 0.009). Increasing severity of obesity was not associated with nocturnal non-dipping.

## Discussion

Our study confirms the high prevalence of abnormal BP in obese children and adolescents. It also underscores the unreliability of OBP measurement and the need for BP monitoring by APBM.

Using ABPM, 8 of the 82 participants (9.8%) were classified as ambulatory hypertension, of which 75% had severe ambulatory hypertension. Elevated BP was present in 22 of the 82 participants (26.8%). In literature, hypertension prevalence ranges from 3.8 to 24.8% in youth with overweight and obesity [23]. Prevalences of elevated BP up to around 15% are reported in unselected children, and to 20–30% in childhood obesity [27–31]. Elevated BP, or former ‘prehypertension’, has shown to be associated with cardiovascular TOD in adolescents and young adults and may be a risk factor of progressing to sustained hypertension [31–37].

Three participants (3.7%) in our cohort were diagnosed with masked hypertension and four (4.9%) with white-coat hypertension. In literature, masked hypertension prevalence ranges from 7.6% in unselected children [8], to 32.3% in obese children with a non-dipping pattern [38]. White-coat hypertension prevalence ranges from 0.6% in 9–10 year old students [39], to 59% in a group of children referred for persistently elevated casual BP [40]. The divergence observed in the prevalence of masked hypertension and white-coat hypertension is likely caused by measurements in different study populations using different diagnostic criteria [40], and by the choice of the upper limits of normalcy [40, 41]. In our study 38.1% of the subjects with stage 1 or 2 hypertension based on OBP measurement demonstrated less severe elevation on ABPM and were classified as elevated BP, also suggesting a white coat phenomenon.

The clinical significance of masked hypertension in children lies in the potentially increased risk for TOD and future cardiovascular events [8, 42, 43]. The impact of white-coat hypertension in children is far less clear [43]. Although white-coat hypertension in adulthood has been associated with cardiovascular morbidity and mortality and progression to sustained hypertension [44, 45], the published cardiovascular events incidences and all-cause mortality relative risks are only slightly higher compared to normotensive people and significantly below the risks associated with sustained hypertension [46, 47].

In our study, more than 20% of the participants with normal OBP turned out to have either elevated BP or masked hypertension on ABPM. These patients would have been missed if classified by OBP. This may convince hesitating healthcare workers to incorporate ABPM in their standard care for overweight and obese children. Discrepancies between OBP and ABPM have been described before in different pediatric populations [3, 48, 49]. Considering (future) cardiovascular risks in patients with elevated BP or masked hypertension, this underscores the importance of performing ABPM in overweight children, although in some children it could be a challenge to obtain an adequate ABPM.

A high prevalence of abnormal circadian variation was present in our study. Nocturnal hypertension has shown to have significant prognostic implications [20]. In childhood and adolescence, literature on the association between nocturnal dipping and morbidity is scarce, although some studies show that non-dipping may be associated with insulin resistance [50, 51]. In adults, a non-dipping status is associated with cardiac structural alterations and a higher risk of CVD events [52]. Although the suggested scheme for staging of ambulatory BP levels of Flynn et al. incorporates night-time mean BP and BP load, dipping status is not included. As such, dipping status represents an entity that needs separate attention. The high prevalence of abnormal circadian variation in this study, with the associated potential risk for TOD and CVD, confirms the importance of performing ABPM in overweight children in order to detect nocturnal hypertension or a decreased or absent dipping status.

No significant differences in terms of age, gender and ethnicity were observed between the different ABPM categories, perhaps due to small sample size. Previous studies noted that ambulatory BP is affected by sex and ethnicity [53, 54]. A recently published systematic review showed that when age was dichotomized according to puberty, elevated BP and hypertension were more prevalent in older children. This association was not consistent when using age as a continuous variable [53].

A higher BMI is an independent risk factor of high BP in children [53]. In our study participants with ambulatory hypertension had a significantly higher BMI Z-score compared to the normal BP group.

Increased HOMA-IR was present in 39% of the subjects with normal BP in this study. All participants with ambulatory hypertension presented with an elevated HOMA-IR, and almost half of the participants with elevated BP. Moreover, a significantly higher prevalence of metabolic syndrome was detected in children with elevated BP and ambulatory hypertension, as well as in non-dippers, indicating the clustering of other CVD risk factors in overweight subjects with high BP when compared to overweight children with normal BP.

To our knowledge the present study is the first using the BP reference values as presented in the updated Clinical Practice Guideline [23] in an overweight childhood population, to compare with ABPM results. The main strength of our study is the large number of available ABPMs. Despite this, low patient numbers in the different ABPM classification groups made it difficult to study factors associated with the different ABPM diagnoses. A few other limitations need to be addressed. First, despite the widespread use of the 2014 AHA Scientific Statement values in the interpretation of ABPMs, several limitations has been recognized, i.e. with regard to generalizability [20]. Robust, universally applicable normative ABPM data in children and adolescents are lacking. Second, by using the current ABPM classification scheme some subjects remain unclassifiable, limiting the comparability between studies due to divergent solutions with regard to the individual classification of these patients. Third, normative data are based on auscultatory measurements, which may provide different values than measurements obtained by using oscillometric devices or ABPM, as obtaining BP by oscillometry could result in an overestimation of BP values [55].

## Conclusions

This study shows a poor correlation between OBP measurement and ABPM in diagnosing hypertension in our population of children and adolescents with overweight or obesity. ABPM allows us to detect white-coat hypertension, masked hypertension and abnormal circadian variation in BP, such as isolated nocturnal hypertension and blunted dipping. The high prevalence of these abnormal ABPM patterns in our overweight pediatric population emphasizes the usefulness of ABPM as a diagnostic tool. The advantage of ABPM to screen out children with ‘hidden’ abnormal ABPM patterns, keeping in mind the association of abnormal ABPM patterns with TOD and future cardiovascular risk, in our opinion outweighs the limitations of ABPM, i.e. the lack of robust, universally applicable normative ABPM data in children and adolescents.

## Data Availability

The dataset used and analysed during the current study is available from the corresponding author on reasonable request.

## List of abbreviations

ABPM: Ambulatory blood pressure measurement
BMI: Body mass index
BP: Blood pressure
CVD: Cardiovascular disease
DBP: Diastolic blood pressure
HDL-C: High-density lipoprotein cholesterol
HOMA-IR: Homeostasis model assessment of insulin resistance
HTN: Hypertension
IQR: Interquartile range
LDL-C: Low-density lipoprotein cholesterol
MetS: Metabolic syndrome
MH: Masked hypertension
OBP: Office blood pressure
SBP: Systolic blood pressure
TOD: Targed-organ damage
WCH: White-coat hypertension
